# Contrasting regulation of live *Bacillus cereus* No.1 and its volatiles on *Shiraia* perylenequinone production

**DOI:** 10.1186/s12934-022-01897-z

**Published:** 2022-08-23

**Authors:** Rui Xu, Xin Ping Li, Xiang Zhang, Wen Hao Shen, Chun Yan Min, Jian Wen Wang

**Affiliations:** 1 College of Pharmaceutical Sciences, Soochow University, Suzhou, 215123 China; 2 Suzhou Institute for Food and Drug Control, Suzhou, 215104 China

**Keywords:** *Shiraia*, *Bacillus*, Volatiles, Direct contact, Hypocrellin, Co-culture

## Abstract

**Background:**

Fungal perylenequinones (PQs) are a class of photoactivated polyketide mycotoxins produced by plant-associated fungi. Hypocrellins, the effective anticancer photodynamic therapy (PDT) agents are main bioactive PQs isolated from a bambusicolous *Shiraia* fruiting bodies. We found previously that bacterial communities inhabiting fungal fruiting bodies are diverse, but with unknown functions. *Bacillus* is the most dominant genus inside *Shiraia* fruiting body. To understand the regulation role of the dominant *Bacillus* isolates on host fungus, we continued our work on co-culture of the dominant bacterium *B. cereus* No.1 with host fungus *Shiraia* sp. S9 to elucidate bacterial regulation on fungal hypocrellin production.

**Results:**

Results from "donut" plate tests indicated that the bacterial culture could promote significantly fungal PQ production including hypocrellin A (HA), HC and elsinochrome A-C through bacterial volatiles. After analysis by gas chromatograph/mass spectrometer and confirmation with commercial pure compounds, the volatiles produced by the bacterium were characterized. The eliciting roles of bacterial volatile organic compounds (VOCs) on HA production via transcriptional regulation of host *Shiraia* fungus were confirmed. In the established submerged bacterial volatile co-culture, bacterial volatiles could not only promote HA production in the mycelium culture, but also facilitate the release of HA into the medium. The total production of HA was reached to 225.9 mg/L, about 1.87 times that of the fungal mono-culture. In contrast, the live bacterium suppressed markedly fungal PQ production in both confrontation plates and mycelium cultures by direct contact. The live bacterium not only down-regulated the transcript levels of HA biosynthetic genes, but also degraded extracellular HA quickly to its reductive product.

**Conclusion:**

Our results indicated that bacterial volatile release could be a long-distance signal to elicit fungal PQ production. Biodegradation and inhibition by direct contact on fungal PQs were induced by the dominate *Bacillus* to protect themselves in the fruiting bodies. This is the first report on the regulation of *Bacillus* volatiles on fungal PQ production. These findings could be helpful for both understanding the intimate fungal–bacterial interactions in a fruiting body and establishing novel cultures for the enhanced production of bioactive PQs.

**Graphical Abstract:**

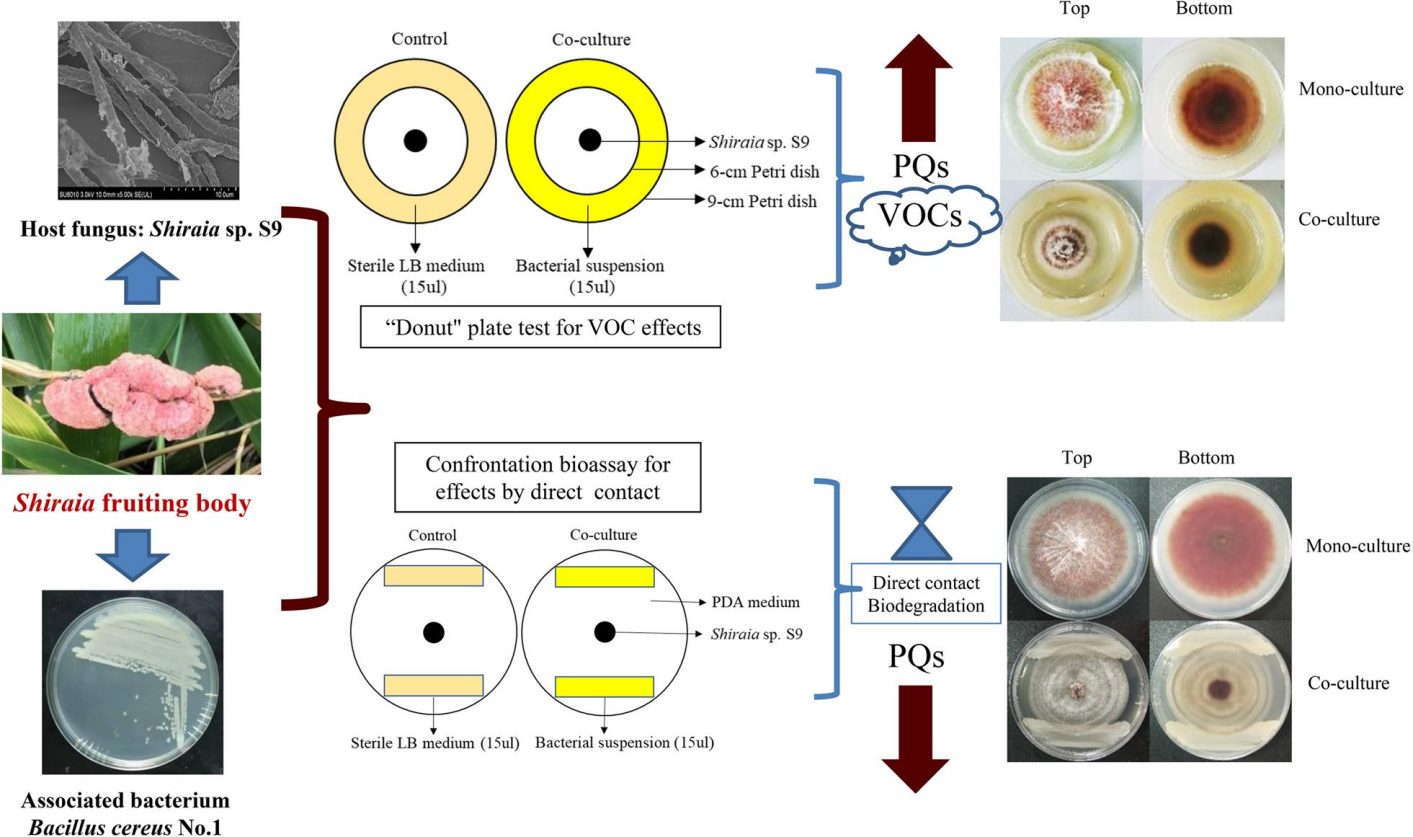

**Supplementary Information:**

The online version contains supplementary material available at 10.1186/s12934-022-01897-z.

## Background

*Shiraia bambusicola* is a medicinally important fungus that parasitizes small bamboo branches and is widely distributed in Southern China and Japan [1]. *Shiraia* fruiting body has been traditionally used in Chinese medicine as “Zhu Huang” for the treatment of rheumatoid arthritis, tracheitis, stomachache and psoriasis [2]. Perylenequinones (PQs) are the main bioactive components from the fruiting bodies, including hypocrellins (HA-HD) and elsinochrome A (EA) [3]. HA and HB have been developing as new non-porphyrin photosensitizers for the photodynamic therapy on cancers [4], virus [5] and multidrug-resistant bacteria [6]. In the natural habitats, diverse bacteria live together with host fungus from the surface and internal tissues of fungal fruiting bodies. Fifty bacteria strains were identified from fruiting bodies of wild Himalayan *Cantharellus* spp. [7]. Fifty-five strains of bacteria including *Bacillus*, *Lysinibacillus*, *Paenibacillus*, *Pandorea*, *Streptomyces*, *Alcaligenes* and *Pseudomonas* existed in the fruiting body of *Agaricus bisporus* [8]. *Pseudomonas fluorescens* was found as the dominant bacterial species in the fruiting body of *Cantharellus cibarius* [9]. In our previous study, a total of 31 strains of bacteria were isolated from *Shiraia* fruiting bodies, whereas *Bacillus* was one of the dominant genera [10].

Most *Bacillus* species are isolated from bulk and rhizosphere soil, and some have been investigated as promising biocontrol agents of plant pathogens. Their efficacy for the plant protection is due to their biosynthesis of various bioactive secondary metabolites [11]. Some *Bacillus* strains released the antifungal cyclic lipopeptides to induce the generation of reactive oxygen species (ROS) and plant systemic resistance against phytopathogenic fungi [12, 13]. *B. amyloliquefaciens* SQR9 could exhibit different antifungal activities by the production of lipopeptides and siderophore bacillibactin [14]. Except such soluble bioactive secondary metabolites, a wide range of volatile organic compounds (VOCs) are also formed in bacilli. Kai (2020) reviewed the diversity and distribution of VOCs from *B. subtilis* [15]. There were totally 231 VOCs produced by 26 strains of *B. subtilis* including hydrocarbons, ketones, alcohols, aldehydes, ester, acids, aromatics and sulfur- and nitrogen-containing compounds. The released volatiles were found to inhibit fungal growth, promote plant growth and induce plant systemic resistance [16, 17]. The VOC benzaldehyde and 1,3-butadiene inhibited significantly the bacterial growth and motility of *Ralstonia solanacearum* and triggered salicylic acid pathway to induce the systemic resistance of tobaccos [18]. The mixture of VOCs produced by endophytic *B. tomentosa* VM11 have biocontrol activity against necrotrophic fungus *Sclerotinia sclerotiorum* via inhibiting mycelial growth and decreasing sclerotia production [19]. Although the high abundance of *Bacillus* was found to be one of the most frequent bacterial communities in fungal fruiting bodies [8, 20], the bioactivity and the physiological roles of *Bacillus* in fruiting bodies were scarcely reported. The spore-forming Bacillaceae were found during the maturation process of *Tuber borchii* fruiting bodies and their bacterial cellulolytic, chitinolytic activities were more likely to be involved in the ascus opening [21]. Xiang et al. (2017) reported that *Bacillus* inside the fruiting body of *A. bisporus* had broad-spectrum antimicrobial activities and potential mushroom growth-promoting abilities [8]. In our previous studies on *Shiraia* fruiting body [10], 31 culturable bacteria were isolated from the fruiting bodies, among which 14 *Bacillus* isolates were found. Illumina high-throughput sequencing results showed that *Bacillus* was the most dominant genus in the fruiting body. All *Bacillus* isolates exhibited various degrees of suppression on fungal red PQ pigments from the mycelium in the confrontation test [10]. Out of 14 isolates, *B*. *cereus* (No.1, No.15)*, B*. *safensis* (No.18) and *B*. *anthracis* (No.19) were able completely to inhibit the fungal HA accumulation. However, there has been so far neither report concerning effects of *Bacillus* strains on the growth of host fungus *Shiraia*, nor regarding its regulation on biosynthesis of fungal PQs. To understand the regulation role of the fruiting body associated *Bacillus* on host fungus, we continued our work to investigate the relationship between *B. cereus* No.1 and host fungus *Shiraia* sp. S9 previously isolated from the fruiting body [10, 22]. This work has been undertaken to compare the effects of live bacterium No.1 and its volatiles on *Shiraia* PQ production. The eliciting role and characterization of the bacterial volatiles were investigated. Based on the impact of VOCs, a novel submerged volatile co-culture of *Shiraia* with *B. cereus* No.1 was established for the enhanced HA production. Simultaneously, the inhibitory mechanism of the live bacterium on HA was also revealed preliminarily. This is the first report on the elicitation role of *Bacillus* VOCs on fungal PQ production.

## Results

### Effects of live *Bacillus* and its volatiles in solid culture

**Fig. 1:**
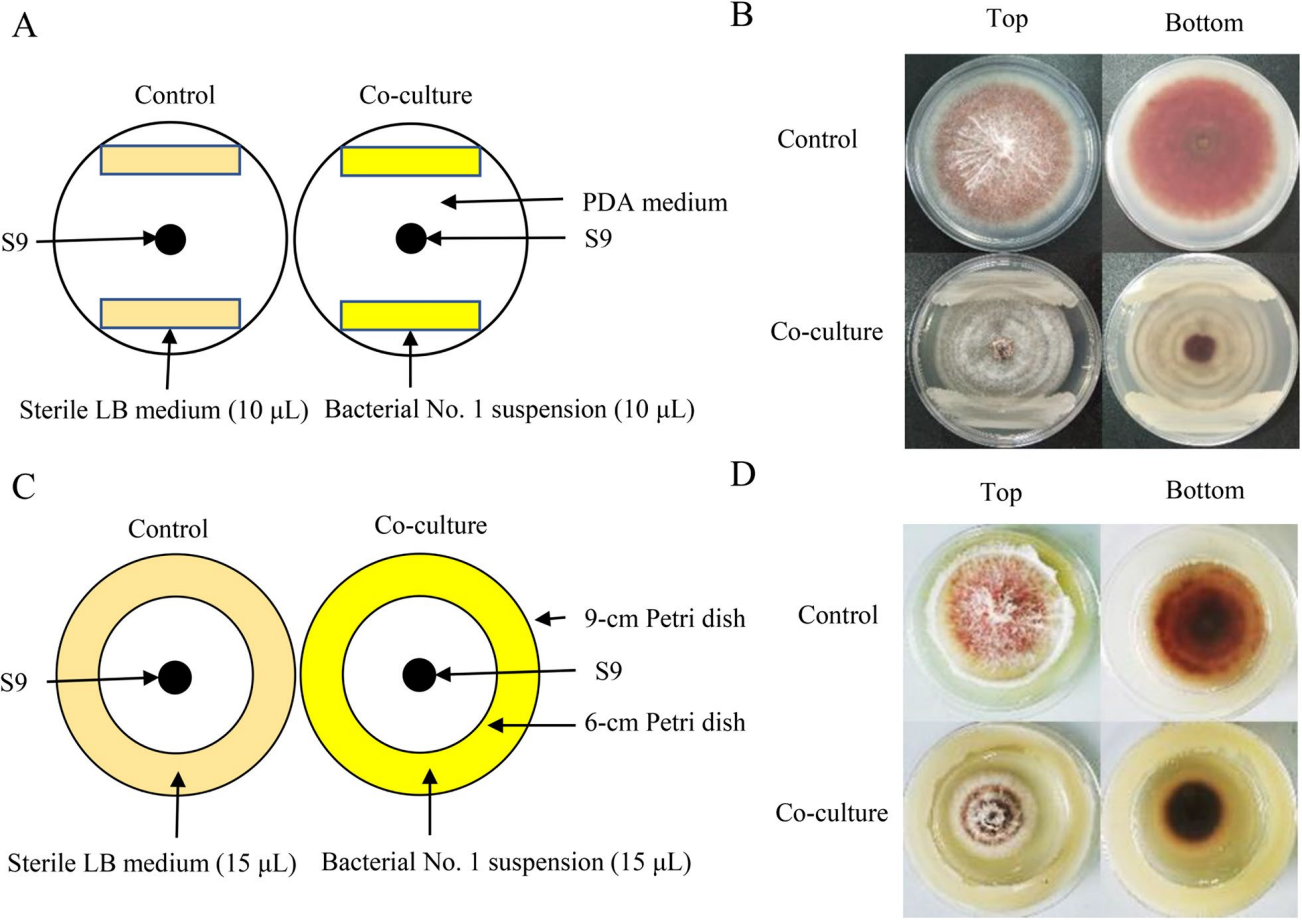
The mode diagrams and plate cultures for the confrontation tests of the live bacterium *Bacillus cereus* No.1 and its host fungus *Shiraia* sp. S9 (**A**, **B**). The mode diagrams and the "donut" plate for the bacterial volatiles and the fungus S9 (**C**,**D**). The cultures were maintained on PDA plate at 28℃ for 8 days

**Fig. 2:**
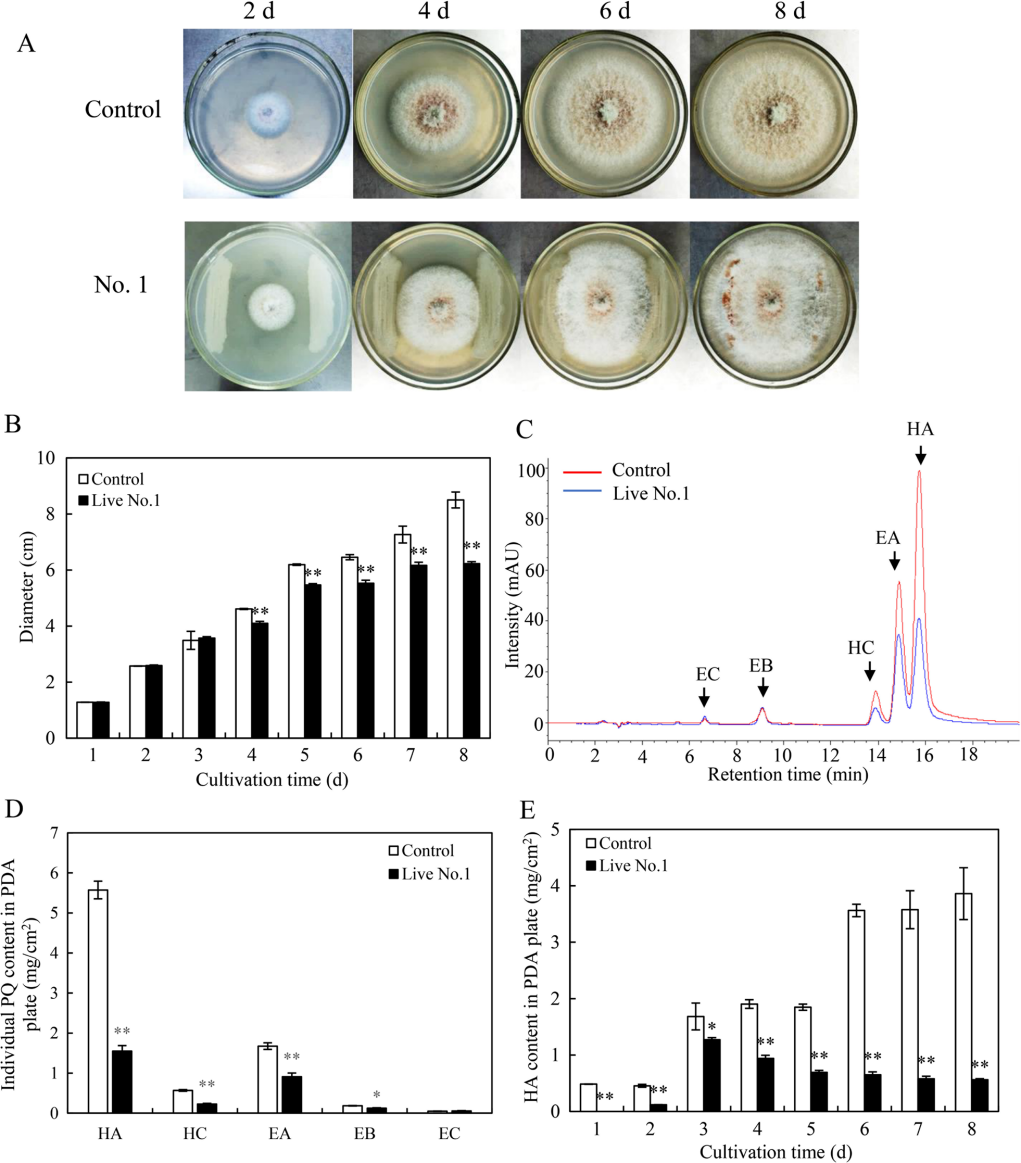
Effects of live *B. cereus* No.1 on the fungal growth (**A**), colony diameter (**B**), individual PQ in HPLC chromatogram (**C**), PQ contents (**D**) and HA content (**E**) of *Shiraia* sp. S9. The co-culture was maintained on PDA plate at 28 °C for 8 days. Values are mean ± SD from three independent experiments. (*p < 0.05, **p < 0.01 versus fungal monoculture as the control group)

An in vitro confrontation bioassay between the host fungus *Shiraia* sp. S9 and bacterium *B. cereus* No.1 were conducted (Fig. 1A). After 8 days of the co-culture, the accumulation of PQ pigments was significantly suppressed by the direct contact (Fig. 1B). The hyphal growth diameter showed an obvious suppression from day 4–8 compared with the control group (Fig. 2A, B). PQs including HA, HC, EA and EB were inhibited under the direct contact of live No.1, about 27.8%, 40.1%, 54.4%, 67.5% decrease of that in the control group (fungal mono-culture) (Fig. 2C, D). Among the PQs, the content of HA was suppressed most significantly (Fig. 2D). The HA content in hyphae was inhibited to the lowest content (0.56 mg/cm^2^) on day 8 (Fig. 2E).

**Fig. 3:**
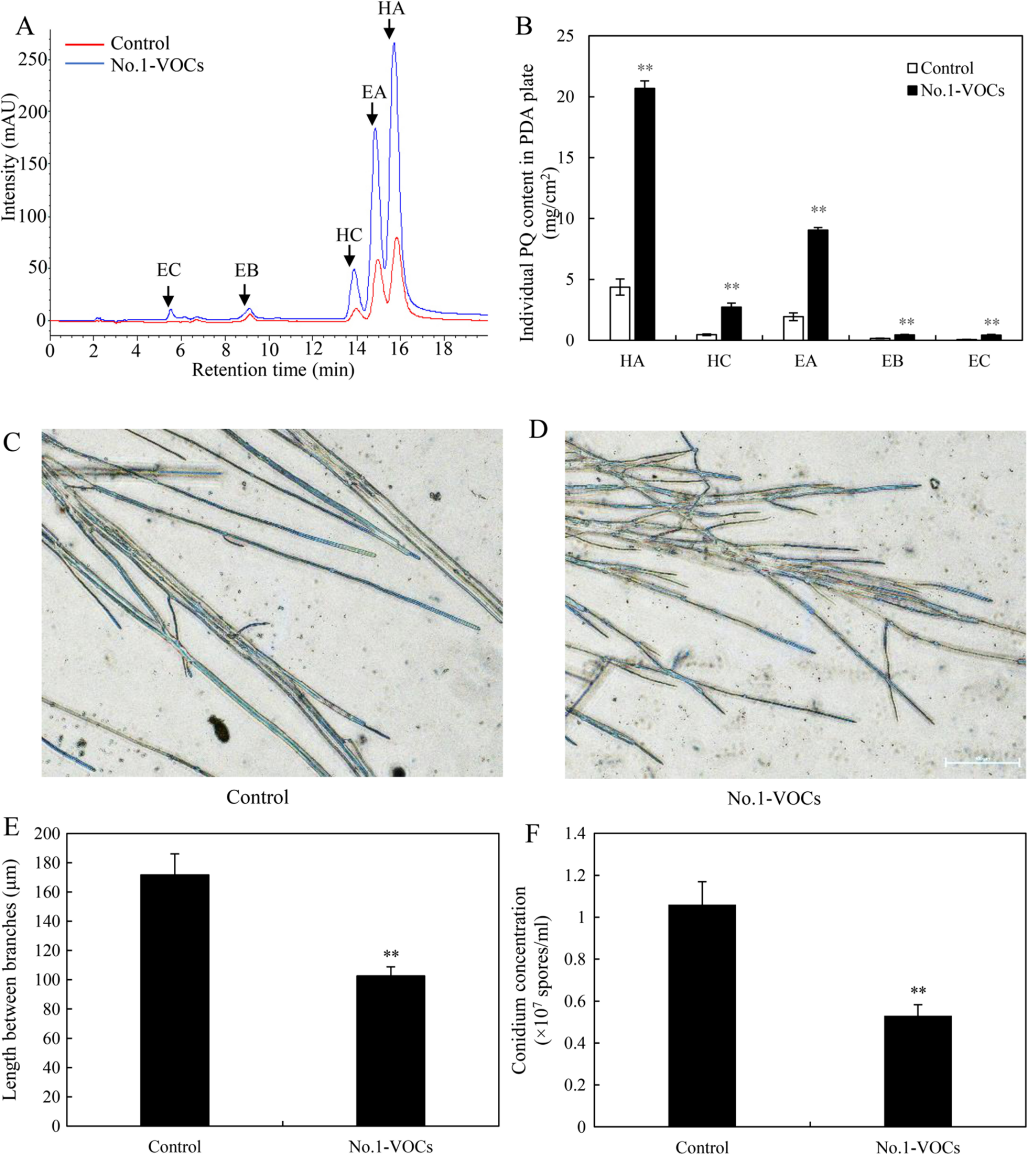
The effects of bacterial volatiles on the fungal growth and PQ production of *Shiraia* sp. S9. The fungus was kept on PDA treated with bacterial volatiles for 7 days. The chromatogram of individual PQs in mycelium (**A**), and the PQ contents in mycelium (**B**) were measured. The mycelial morphology was observed (**C** and **D**, × 400). The length between mycelial branches (**E**) and conidium concentration of S9 were measured on day 7 (**F**). The bacterial suspension and fungal mycelial plugs were inoculated simultaneously. Values are mean ± SD from three independent experiments. (**p < 0.01 versus fungal monoculture as the control group)

A "donut" plate assay (Fig. 1C) was conducted for the investigation of the bacterial volatile-mediated effects on fungus S9. It was an interesting finding that the bacterial volatiles could promote the secretion of red PQ pigments in the plates (Fig. 1D). The contents of PQs including HA, HC, EA, EB and EC were enhanced significantly, about 4.74-, 4.67-, 5.93-, 2.81-, 7.00-fold higher than that of the control group, respectively (Fig. 3A, B). However, the bacterial volatiles shortened the distance of hyphal branches (Fig. 3C–E) and suppressed fungal conidiation (Fig. 3F).

### Identification of bacterial VOCs and their effects on fungal HA production

**Fig. 4:**
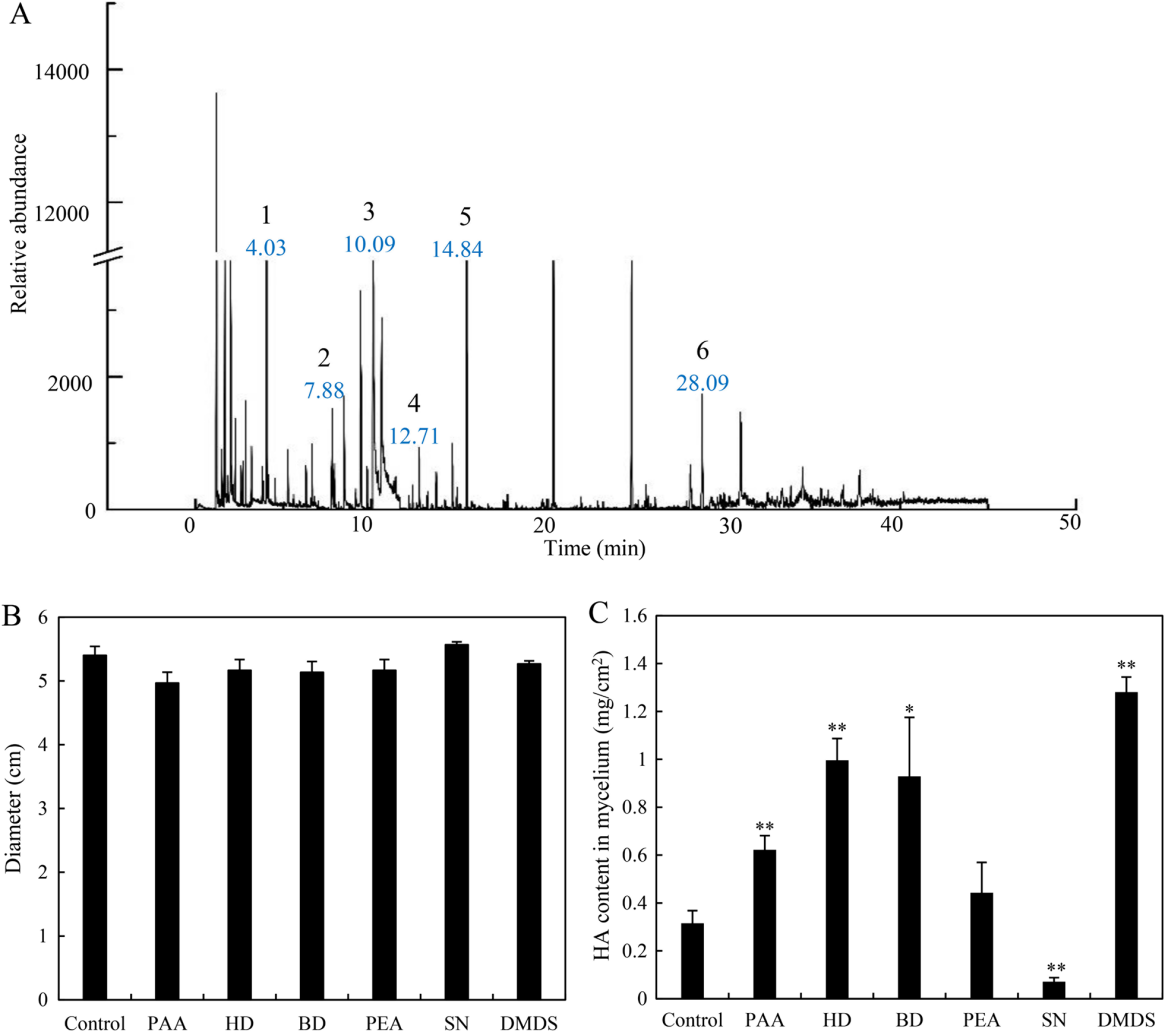
The VOC identification and their effects on the fungus S9. The total ion current diagram of VOCs from No.1 after 24-h culture (**A**). Main peaks: 1, dimethyl disulfide (t_R_ 4.03 min); 2, styrene (t_R_ 7.88 min); 3, benzaldehyde (t_R_ 10.09 min); 4, phenylacetaldehyde (t_R_ 12.71 min); 5, phenylethyl alcohol (t_R_ 14.84 min); 6, hexadecane (t_R_ 28.09 min). Effects of main VOCs on fungal growth diameter (**B**) and HA content (**C**). Different VOC standards were added at 5 mg/mL on day 3 of the plate cultures of fungus S9 at 28 ℃ and the measurement was token after 2 days of the treatment. VOCs such as phenylacetaldehyde (PAA), hexadecane (HD), benzaldehyde (BD), phenylethyl alcohol (PEA), styrene (SN) and dimethyl disulfide (DMDS) were selected. Values are mean ± SD from three independent experiments. (*p < 0.05, **p < 0.01 versus fungal monoculture as the control group)

A total of 34 VOCs were detected from *B. cereus* No.1 by GC–MS (Fig. 4A) and listed in Table 1 for 13 compounds (a relative area of more than 1.0% and more than 70% identity), and Additional file 1: Table S1 for other compounds (a relative area of less than 1.0% or less than 70% identity). The most abundant volatile metabolites were identified as ketones, alkanes, aldehydes, esters, alcohols and sulfides. The tested commercial VOCs at 5 mg/mL exhibited no significant influence on the fungal growth (Fig. 4B), but fungal HA production was promoted markedly by most of them (phenylacetaldehyde, dimethyl disulfide, phenylethyl alcohol, hexadecane and benzaldehyde) (Fig. 4C). The content of HA in hyphae reached 1.28 mg/cm^2^ on day 5, about 4.06 times that of the control group under the treatment of dimethyl disulfide.

**Table 1:** The analysis of VOCs from strain No.1 by headspace solid phase microextraction-gas chromatography-mass spectrometry (HS–SPME–GC–MS)

Compound ^a^	Identity (%)^b^	Peak area (%)^c^	RT^d^	MW^e^	MF^f^
Methylene chloride 2-Butanone Dimethyl silanediol Dimethyl disulfide S-methyl ester butanethioic acid Styrene 2,5-Dimethyl-pyrazine Phenylacetaldehyde (Bromomethyl)-silane (e)-2-Nonen-1-ol Phenylethyl alcohol Hexadecane Tetracosane	94 72 83 96 78 91 83 97 83 78 74 72 90	7.82 11.59 1.49 11.90 3.04 1.21 3.11 24.96 1.51 1.00 1.55 1.72 3.66	1.665 1.99 2.855 4.032 7.766 7.875 8.441 12.328 13.67 14.58 14.841 28.099 30.962	84.93 72.11 92.17 94.2 118.2 104.15 108.14 106.12 120.15 136.19 122.16 226.44 338.65	CH_2_Cl_2_ C_4_H_8_O C_2_H_8_O_2_Si C_2_H_6_S_2_ C_5_H_10_OS C_8_H_8_ C_6_H_8_N_2_ C_7_H_6_O C_8_H_8_O C_8_H_12_N_2_ C_8_H_10_O C_16_H_34_ C_24_H_50_

^a^The tested compounds with a relative area of more than 1.0% and more than 70% identity are listed

^b^Spectra similarity of analyte compounds with those available in the spectral library (NIST)

^c^Relative area of detected compounds as a percentage in reference to the total spectra peaks

^d^Retention time (RT) of the compounds in the GC–MS analysis

^e^Molecular weight (MW) of detected compounds

^f^Molecular formula (MF) of identified compounds

### Effects of the bacterium on *Shiraia* growth and HA production in liquid culture

**Fig. 5:**
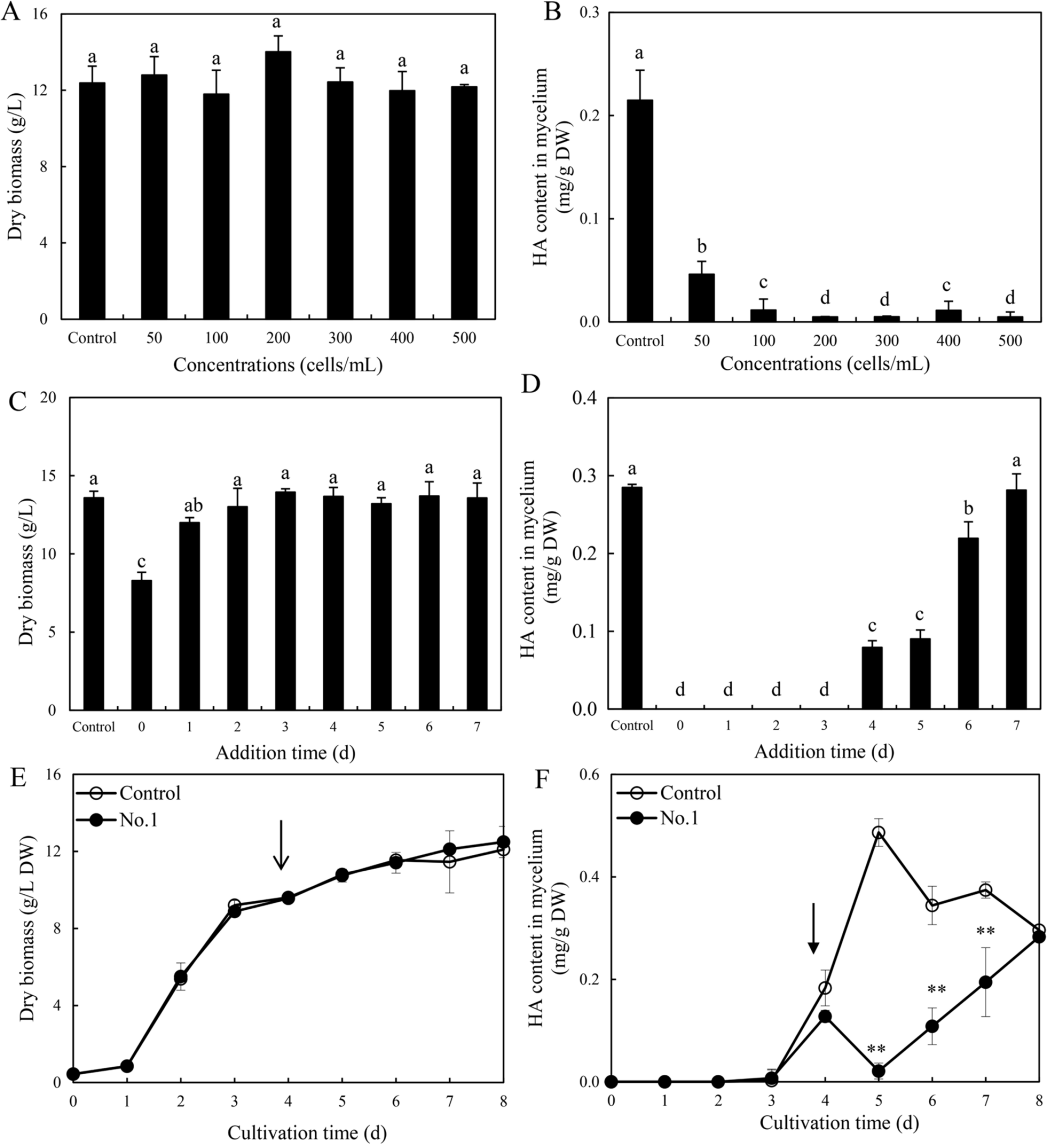
Effects of live bacterium No.1 at 50–500 cells/mL on fungal biomass (**A**) and HA content (**B**) in liquid cultures. Live No.1 was added on day 3 of the mycelium culture (8 days). The effects of different adding time (day 0–7) on fungal biomass (**C**) and HA content (**D**) were also measured. Time profiles of fungal biomass (**E**) and HA content in mycelium (**F**) under the bacterial treatment. Live No.1 at 500 cells/mL was added on day 4 of the mycelium culture (8 days). The *arrow* represents the addition time of live No.1. Values are mean ± SD from three independent experiments (*p < 0.05, **p < 0.01 versus fungal monoculture as the control group). Different letters above the bars mean significant differences (*p* < 0.05)

We continued to investigate the effects of the live bacterium on HA production in mycelium culture. After the application of bacterium No.1 at 50–500 cells/mL to 3-day-old fungal cultures, the fungal biomass was not altered during the culture (Fig. 5A). The content of HA was suppressed markedly by live bacterium at a density of 50–500 cells/mL (Fig. 5B). When No.1 at 500 cells/mL was added, the fungal biomass was suppressed only under the addition on day 0 and 1 (Fig. 5C). The addition of the bacterium in early stages (day 0–3) caused the full suppression of HA production (Fig. 5D). We chose the addition at 500 cells/mL on day 4 of initial culture for the time-course investigation. We found the addition on day 4 had no effects on the fungal biomass (Fig. 5E), whereas the accumulation of HA dropped rapidly to 0.02 mg/g dry weight (DW) after 24 h, but rose to the control level gradually (Fig. 5F).

**Fig. 6:**
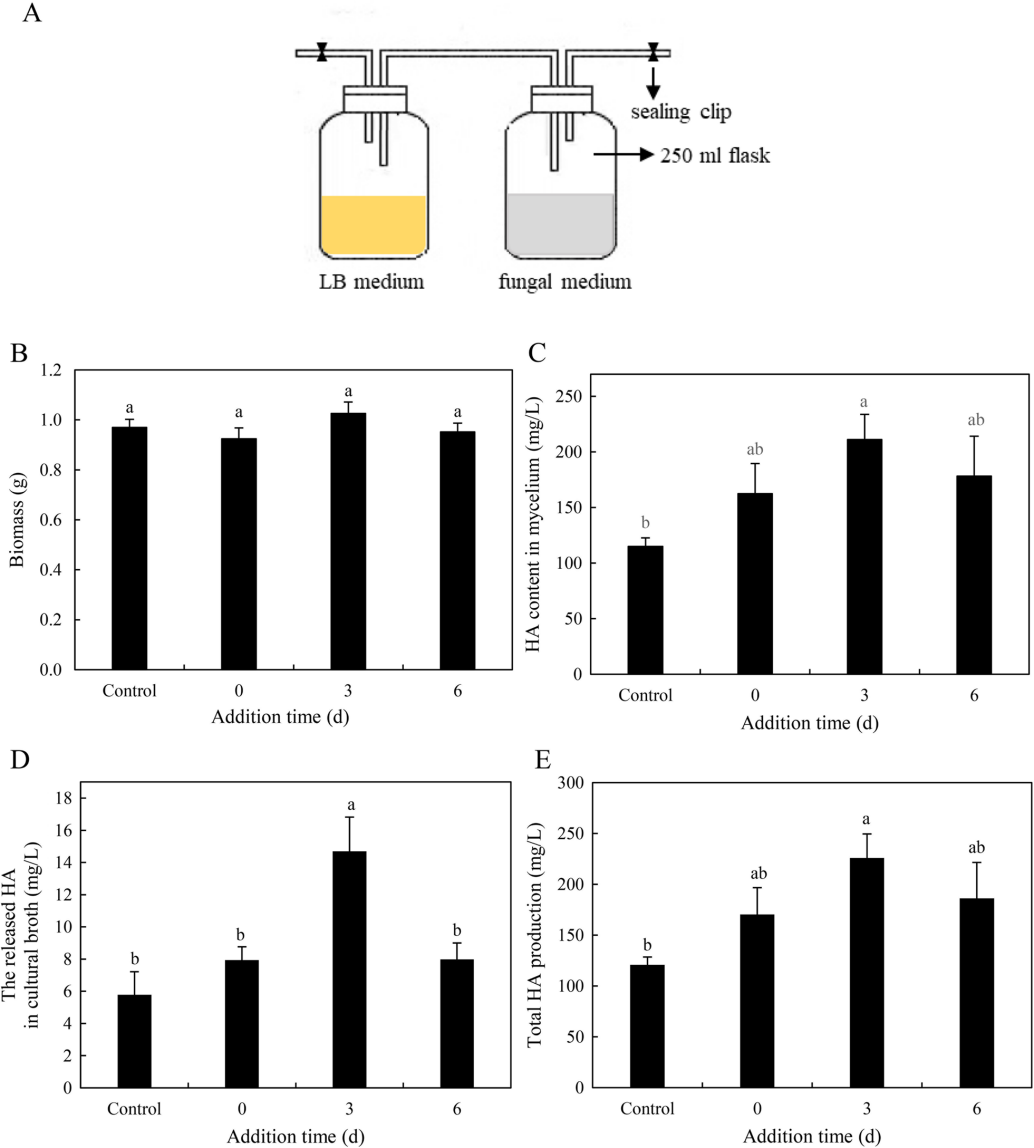
Effects of bacterial volatiles on fungal growth and HA production in the submerged volatile co-cultures (**A**). The fungal dry biomass (**B**), HA contents in mycelium (**C**), the released HA in cultural broth (**D**) and total HA production (**E**) were measured in the culture. Two culture flasks were connected through sealed glass tube. The culture was maintained in 250-mL flask containing 100 mL of the liquid medium at 150 rpm and 28 ℃ for 8 days. An equal volume of sterile LB broth instead of bacterial suspension added to flask was used as control group. Values are mean ± SD from three independent experiments. Different letters above the bars mean significant differences (*p* < 0.05)

To analyze the influence of the bacterial VOCs on HA production in liquid culture, a submerged volatile co-culture was established (Fig. 6A). In this culture system, the flask containing bacterial suspension at 500 cells/mL was connected with fungal culture on day 0, 3, 6. The fungal biomass of the culture was not altered (Fig. 6B). However, the treatment could not only promote HA production in the mycelium culture (Fig. 6C), but also facilitated the release of HA into the medium after the connection on day 3 (Fig. 6D). The total production of HA reached the maximum 225.9 mg/L on day 8, about 1.87 times that of the control group (Fig. 6E).

### Degradation of HA by live bacterium

**Fig. 7:**
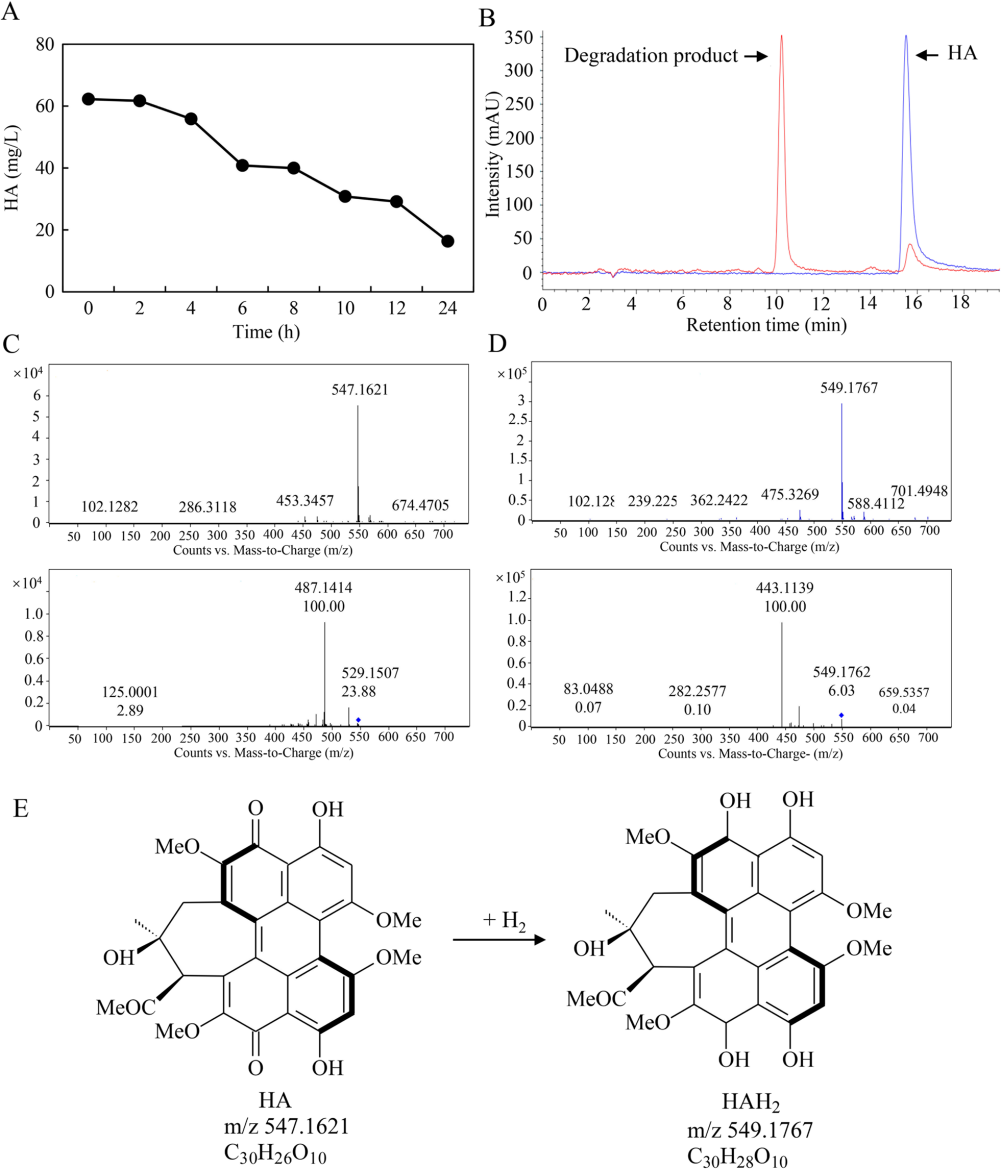
The degradation of HA by *B. cereus* No.1. The degradation kinetic (**A**) and HPLC chromatogram (**B**) of standard HA by the bacterium. MS and MS^2^ spectra scan relative to HA standardat 40 mg/L (**C**) and its degradation products treated with live bacterium No.1 after 48 h (**D**). Structures of HA and a possible degradation product HAH_2_ (**E**). The live bacterial No.1 was added to the culture medium containing HA (40 μg/mL) and kept at 28 ℃ in the dark at 150 r/min for 2 days

In order to find out the reason why fungal HA was decreased in the presence of live bacterium, we applied live No.1 to culture medium containing standard HA (Fig. 7A). Live bacterium at 500 cells/mL could rapidly reduce HA from the initial content 62.3 mg/L to 16.3 mg/L in the medium within 24 h, and the percentage of HA degradation was approximately 74.8%. HA degradation by live No.1 was also confirmed in the HPLC chromatogram (Fig. 7B), where a new peak for the degradation product was found at the retention time of 10.2 min. The possible degradation product in the medium was identified by UPLC-Q-TOF–MS/MS. Compared with the standard HA with a molecular formula of C_30_H_26_O_10_ with [M + H] ^+^ ion peaks at m/z 547.1621 (Fig. 7C), the suggested molecular formula of degraded product was C_30_H_28_O_10_ with [M + H] ^+^ ion peaks at m/z 549.1767 (Fig. 7D). The results suggested that the HA could be hydrogenated and reduced by live No.1 into HAH_2_ (Fig. 7E).

### Effects of bacterial components on *Shiraia* growth and HA

**Fig. 8:**
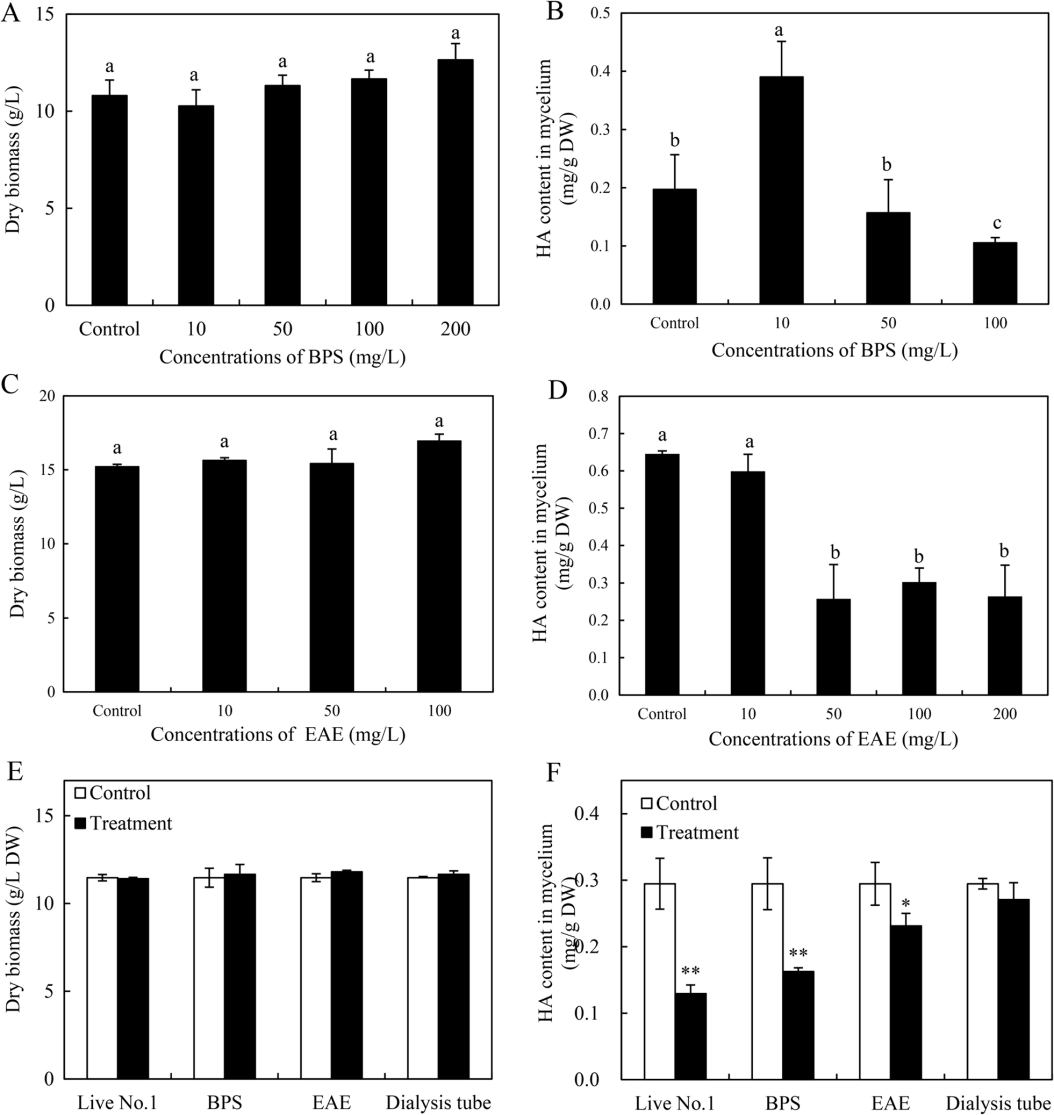
Effects of live bacterium of *B. cereus* No.1 and the bacterial components on fungal growth and HA production of *Shiraia* sp. S9. The effects of different concentration of crude polysaccharides (BPS) and ethyl acetate extract (EAE) on fungal biomass (**A**, **C**) and HA contents in the mycelium (**B**, **D**). Effects of live bacterium at 500 cells/mL, BPS at 100 mg/L, EAE at 200 mg/L and bacterium in the dialysis tubing on fungal biomass (**E**) and HA contents (**F**) in mycelium culture. The live No.1, EAE, BPS and bacterium in the dialysis tubing were added on day 3 of the 8-day-culture of the fungus S9. Fungal monoculture was used as the control group. Values are mean ± SD from three independent experiments (*p < 0.05, **p < 0.01 versus control groups). Different letters above the bars mean significant differences (*p* < 0.05)

Four different bacterial components including bacterial ethyl acetate extract (EAE), crude bacterial polysaccharide (BPS), bacterium No.1 in dialysis tubes and live bacterium were applied to *Shiraia* mycelium culture on day 3. The different concentrations of EAE and BPS were compared (Fig. 8A–D). The results revealed that these extracts had no effect on the fungal biomass (Fig. 8A, C), but EAE ≥ 50 mg/L and BPS at 100 mg/L could suppress significantly the mycelial content of HA (Fig. 8B, D). Although the fungal biomass was not altered in the cultures under the various treatments (Fig. 8E), the live No.1 and bacterial extracts (EAE and BPS) in the cultures suppressed the HA biosynthesis (Fig. 8F). The inhibitory effect of live No.1 on HA was the most significant and the content of HA was reduced by 56.2% to 0.13 mg/g DW.

### Effects of No.1 on key gene expression for HA biosynthesis

**Fig. 9:**
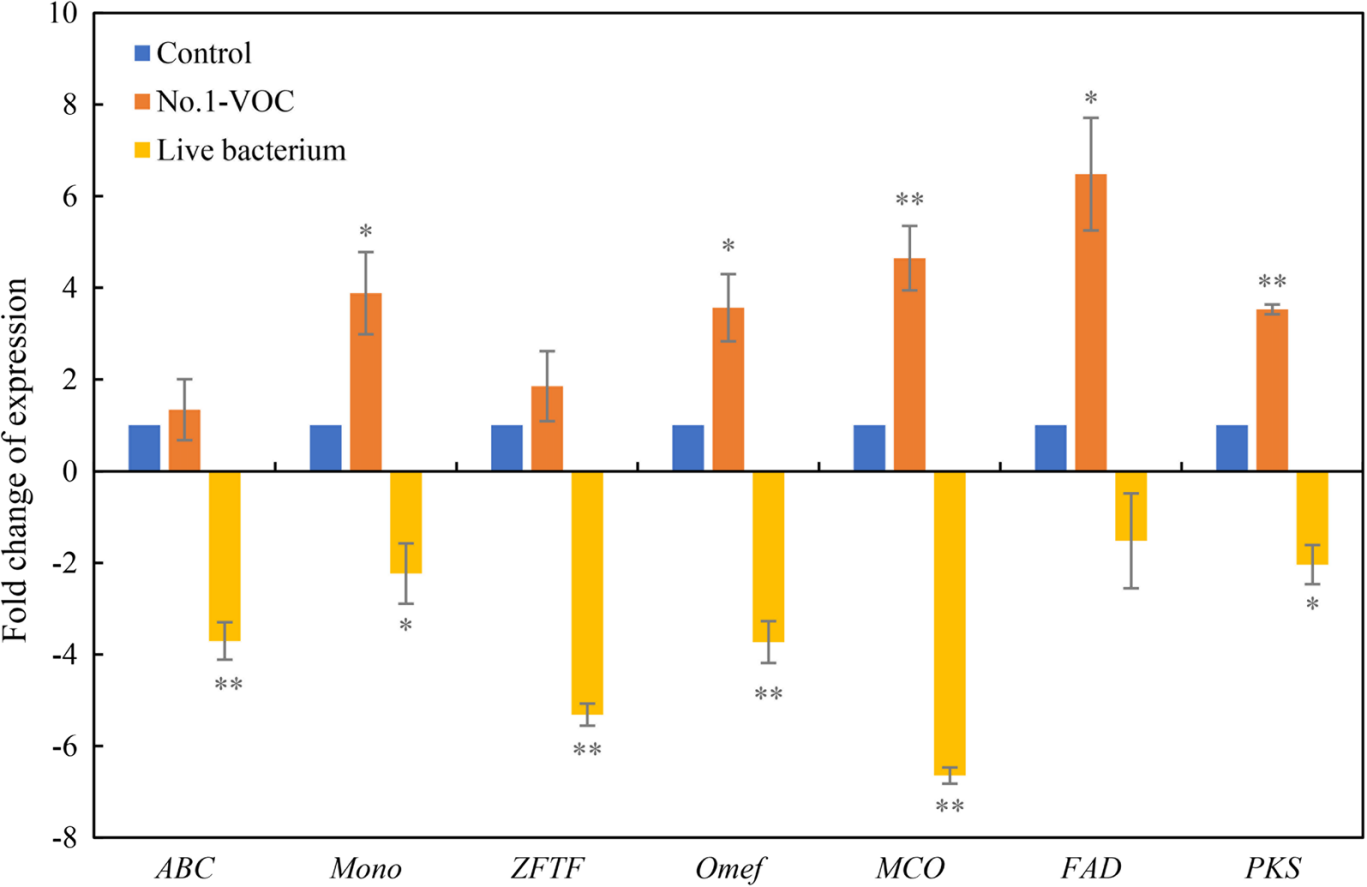
Effects of live *B. cereus* No.1 and its volatiles on the expression of HA biosynthetic genes of *Shiraia* sp. S9 in solid culture. The confrontation tests between live bacterium or bacterial volatiles with the fungus S9 were the same as specified in Fig. 1. The cultures were maintained on PDA plate at 28℃ for 8 days. Values are mean ± SD from three independent experiments. (*p < 0.05, **p < 0.01 versus fungal monoculture as the control group)

After 8 days of the treatments including live bacterium and bacterial volatiles in the plates, seven HA biosynthesis-related genes including multicopper oxidase (*MCO*), polyketide synthase (*PKS*), monooxygenase (*Mono*), FAD/FMN-dependent oxidoreductase (*FAD*), *O*-methyltransferase (*Omef*), zinc finger transcription factor (*ZFTF*) and ATP-binding cassette (*ABC*) were quantified by using qRT-PCR (Fig. 9). The expression of all genes related to HA biosynthesis were down-regulated by live bacterium No.1 in direct contact, about 6.6-, 2.0-, 3.7-, 1.5-, 2.2-, 5.3- and 3.7-fold of the control group, respectively. On the other hand, the bacterial volatiles showed an opposite way on the expression of HA biosynthetic genes, up-regulating the expression of most tested genes including *Mono, Omef, MCO*, *FAD* and *PKS*.

## Discussion

*Shiraia* fruiting bodies are main resources for Chinese folk medicine (Zhu Huang) and the important photosensitizer hypocrellins for photodynamic therapy [23]. *Shiraia*-associated microbial communities are rich and diverse in the fruiting bodies or inside bamboo tissues. The associated fungi were isolated from the fruiting bodies, shoots and leaves of fresh bamboos (*Phyllostachys* sp., *Sasa* sp. and *Brachystachyum densiflorum*) [24–27]. Yan et al. (2021) isolated 17 endophytic fungi from the bamboo branches with fruiting bodies of *S. bambusicola* and the endophytic *Arthrinium* sp. AF-5 was found to stimulate mycelial growth and hypocrellin production of *S. bambusicola*. [28]. *Phoma* sp. BZJ6, an endophytic fungus could promote laccase from *S. bambusicola* [27]. In the fruiting bodies, the host *Shiraia* and bacteria were found living together [10] and one of fruiting body-associated bacteria *P. fulva* SB1 stimulated fungal PQ production [22]. These results indicated that *Shiraia*-associated microbes may develop intimate interactions with *Shiraia* on regulation of the fungal metabolites. In this study, the live dominant bacterium *B. cereus* No.1 and its volatiles exerted the contrasting effects on *Shiraia* PQ production. The contents of PQs including HA, HC and EA-EC were increased by about 2.81–7.00-fold under the treatment of bacterial volatiles (Fig. 3A, B), whereas the live bacterium in direct contact had inhibitory effects on fungal PQ production in both the confronting test (Fig. 2) and the mycelium co-cultures (Fig. 5). To date, there is scarce report on volatile-mediated promotion and the contacting suppression by the same bacterium on fungus. *P. aeruginosa*, the most frequently colonizing bacterium in chronic lung infection was associated with the fungal pathogens *Aspergillus fumigatus*. The bacterium was found to inhibit conidial germination and biofilm formation *of A. fumigatus* by direct contact [29]. However, bacterial VOCs released by *P. aeruginosa* had the stimulatory effects on the fungal growth [30]. We reported for the first time that the volatiles released by *Bacillus* had stimulatory effects on fungal PQ production. Since the dominant bacterium *B. cereus* No.1 and the host fungus *Shiraia* sp. S9 shared the same habitat in the fruiting bodies, these results suggested the existence of competitive or even more complex interactions between the bacteria and fungi in fruiting bodies.

HA, the main hypocrellin from *Shiraia* fruiting bodies has a photodynamic antibacterial activity against both Gram-positive *Staphylococcus aureus*, *B. subtilis* and Gram-negative *Escherichia coli*, *Salmonella typhimurium* [31, 32]. The antibacterial activities are contributed by HA-induced ROS targeting to bacterial cell wall and membranous enzymes. HA was synthesized in *Shiraia* cells, but could be released to extracellular space [22], leading to the bacterial inability to grow around the host fungi. Usually, bacteria release VOCs as defensive weapons to antagonize fungi at a long distance [33]. Inhibition on mycelium growth or spore germination was reported most frequently by bacterial VOCs [19, 34]. In our study, *Shiraia* conidiation was suppressed and the distance of hyphal branches was decreased by volatiles of *B. cereus* No.1 (Fig. 3), indicating a slight stress applied to the fungus. The volatiles triggered the upregulation of various genes of PQ biosynthesis gene cluster (Fig. 9), including *FAD*, *MCO*, *Mono*, *Omef* and *PKS* [35, 36]. The enhanced transcript levels were responsible for the induced PQs (HA, HC and EA-EC) by the volatiles. The main VOCs from the bacterium are aldehydes, ketones, alkanes, sulfides, esters and alcohols (Table 1 and Additional file 1: Table S1). Some VOCs such as phenylacetaldehyde, dimethyl disulfide, phenylethyl alcohol, hexadecane and benzaldehyde showed the significant promotion on the fungal HA accumulation (Fig. 4C). The VOCs were proved to be responsible for the unexpected stimulatory effects on *Shiraia* hypocrellins. These results also demonstrated that the fungus may boost up its antibacterial activity via the accumulation of PQs in response to the bacterial volatile-mediated antagonism. On the other hand, this bacterial-fungal interaction could be harnessed to improve biotechnological production of important fungal metabolites. Based on the stimulatory effects of VOCs by edible mushroom *Pleurotus ostreatus* on exopolysaccharide production of the medicinal fungus *Ganoderma lucidum*, Asadi et al. (2021) established a novel submerged volatile co-culture system in which the specific yield of exopolysaccharides was increased about 2.2-fold by 6-day treatment of *P. ostreatus* VOCs [37]. Currently, versatile systems for microbial mono- and co‑cultures have been constructed for production of value‑added compounds [38, 39]. In this study, we also made an effort to establish the submerged volatile co-culture system (Fig. 6). This is the first report on the use of bacterial volatiles as a novel elicitor to simulate PQ production in mycelium cultures. With the optimization on medium components, culture condition and other bioprocess parameters, the production of fungal metabolites in the submerged volatile co-culture system could be enhanced greatly.

In contrast to the enhanced *Shiraia* PQ production by the elicitation of the bacterial VOCs, significant inhibition on PQ production was observed in situations of direct contact of live bacterium No.1 and *Shiraia* sp. S9 in both solid plates (Fig. 2) and mycelium cultures (Fig. 5). The suppression on the fungal growth was obvious by direct contact with the live bacterium after day 4 on the cultural plates (Fig. 2B). The bacterial extracts (BPS and EAE) were also proved to have the inhibitory effects on fungal HA production in the mycelium culture (Fig. 8), suggesting that not only direct contact but some bacterial cell components could contribute to the suppression. In our study, the expressions of all tested genes for PQ biosynthesis were down-regulated by live bacterium in solid culture (Fig. 9). This is in agreement with the finding that *B. megaterium* inhibited fungal aflatoxin and cyclopiazonic acid production of *A. flavus* through down-regulating the pathway gene expression [40]. On the other hand, live bacterium No.1 could quickly degrade HA in the culture medium by 74.8% within 24 h (Fig. 7A) and conversed it to a possible HAH_2_ by the transfer hydrogenation reduction (Fig. 7C–E). Therefore, it can be suggested that the bacterial degradation capacity was linked to the antagonistic action of the bactericidal HA produced by *Shiraia.* The bacterial degradation mechanism and degraded metabolites need further investigation. As photoactivated PQ toxins are involved in the fungal pathogenicity of *Cercospora*, *Mycosphaerella*, and *Alternaria* in plant diseases [41], our results also indicated that the *B. cereus* No.1 hold a promise as potential biocontrol agent due to its inhibition of PQ biosynthesis and its biodegradation capacity.

## Conclusion

In summary, this study presented the first assessment of dual effects of a fruiting body-associated bacterium (*B. cereus* No.1) on metabolite production (PQs) of host fungus: promotion by bacterial VOCs and inhibition by direct contact. *Shiraia* fruiting bodies host a wide diversity and high abundance of bacteria and produce pharmaceutically and agriculturally important PQs such as hypocrellins and elsinochromes. It is an interesting finding that VOCs from one of the predominant *Bacillus* strains (No.1) stimulated PQ biosynthesis via transcriptional regulation. The study showed unexpectedly that host fungus could interact with the associated bacteria by PQ production at a distance via sensing bacterial VOCs. Based on this impact of VOCs, a novel submerged bacterial volatile co-culture was constructed for biotechnological production of the important photosensitizer HA. In contrast, the live bacterium not only down-regulated the transcript levels of HA biosynthetic genes, leading to suppression on HA accumulation by direct contact, but also degraded extracellular HA quickly. Although bacterial signal compounds and VOCs perception mechanisms in fungus need to be further elucidated, our study provided a new understanding of specific cues of bacterial VOCs in bacteria-fungi interactions. These findings could be harnessed for the potential biotechnological application of VOCs in submerged co-culture for PQ production and agricultural application for the live bacterium as a biocontrol agent for plant diseases.

## Material and methods

### Strains, media and culture conditions

The PQ-producing strain *Shiraia* sp. S9 and its associated bacterium *B. cereus* No.1 used in this study were isolated in our Lab [10] and deposited at the China General Microbiological Culture Collection Center (CGMCC) with registered number CGMCC16369 and 21176, respectively. The strain of *Bacillus* (*B. cereus* No.1) selected for this study was based on our previous report [10] for the inhibition of hypocrellin production by bacteria associated with the fruiting body. The physiological, biochemical characteristics and 16S rDNA gene sequence analysis were used for the identification of the stain [10]. The phylogenetic tree of *B. cereus* No.1 was presented in Additional file 1: Fig. S1. The fungus strain S9 was stored at 4 °C on a potato dextrose agar (PDA) plate and bacterial strain No.1 was conserved on Luria–Bertani (LB) agar plate at 4 °C. To initiate the liquid culture, S9 was cultured on a PDA plate in the dark for 8 days at 28 °C. The seed culture preparation, the medium component and conditions for the liquid cultures were in the same as described in our previous report [42]. Then, 5 mL of the seed broth was transferred into a 150-mL flask with 50 mL liquid medium and was incubated at 28 °C and 150 rpm on a rotary shaker (ZD-8802, Hualida, Suzhou, China).

### Co-cultures of live bacterium and its volatiles with *Shiraia* sp. S9

The confrontation bioassay in vitro between the fungus S9 and the bacterial strain No.1 was conducted according to our previously reported method [22]. Mycelial plug (5-mm-diameter) was taken from the margin of 8-day-old culture and placed on the center of a 90 mm PDA Petri dish (Fig. 1A). After 24 h of incubation, 10 μL of bacterial suspension (10^5^ cells/mL) was streaked in parallelly on both sides of the PDA, approximately 7 cm apart from each other. The equivalent LB broth without the bacterium was used as the control group. The “donut” plate assay was used to measure the effects of bacterial VOCs on the fungus with a slight modification [30]. A smaller 60-mm petri dish lid was placed upside down in a 90-mm petri dish to separate physically the bacterial media in the outside ring and fungal media in the inside (Fig. 1C). A volume of 12 mL LB media was pipetted into the outer ring and 10 mL of PDA media was added to the inside. The bacterial suspension (10^5^ cells/mL) of 15 μL after 24 h of cultivation was inoculated on LB medium and the fungal mycelial plugs of 5-mm-diameter cultured for 8 days were inoculated on the PDA medium simultaneously. The diameter of fungal growth and HA content were measured when the co-culture was kept for 2–7 days at 28°C in a constant temperature incubator (GNP-9080BS-III, CIMO, Shanghai, China).

The submerged volatile co-culture (Fig. 6A) was established to determine the effects of bacterial VOCs on the fungus in liquid culture according to the report by Asadi et al. [37]. This co-culture system was composed of a 250 mL culture flask for fungus S9 and one 250-mL culture flask for bacterium containing 100 mL LB broth. Two flasks were connected through a glass tube. The S9 seed culture (10 mL) was added into a 250-mL flask with 100 mL liquid medium. After 24 h of cultivation at 37°C, bacterial suspension at a final density of 500 cells/mL was added to the flask for bacterial volatile producer on day 0, 3, 6 of the fungal culture and connected to the flask for mycelial culture. Then, the co-cultures were kept on a rotary shaker at 150 rpm at 28 °C for 8 days. The equivalent volume of fresh LB broth without inoculating bacterium was added to the flask as a control.

### Elicitation of bacterial components on fungal PQ production

To compare effects of bacterial extracts and live bacterium on fungal PQ production of *Shiraia* sp. S9, ethyl acetate extract (EAE) from the bacterial fermentation broth, bacterial polysaccharides (BPS) and live bacterium in dialysis tubes were prepared according to our previous study [22]. The mycelium cultures were added with live bacteria No.1 (500 cells/mL), EAE (10–200 mg/L), BPS (10–100 mg/L), and 2 mL of live bacteria in dialysis tubes (5 × 10^3^ cells/mL), respectively on day 3 and kept on a rotary shaker at 150 rpm at 28 °C for another 5 days. The mono-culture of S9 treated with the equal volumes of sterile LB broth was used as the control group.

### Analysis of bacterial VOCs

VOCs from *B. cereus* No.1 after 24 h of incubation at 28℃ were collected and analyzed as described by Li et al. (2010) [43]. Briefly, the VOCs were determined by headspace solid-phase microextraction (HS-SPME) coupled with gas chromatography-mass spectrometry (GC–MS, Agilent 6890N-5975B, American) equipped with an HP-5MS column (Agilent, CA, USA). Mass spectra were obtained using the scan modus (total ion count, 50–500 m/z). Compounds were identified by comparison of mass spectra and retention times with those of available standards in the library of the National Institute of Standards and Technology (NIST).

### PQ extraction and quantification

The fungal PQs in PDA and mycelium culture of *Shiraia* sp. S9 were extracted according to our previous study [44]. The PQ contents were measured by the reverse-phase Agilent 1260 HPLC system (Agilent Co., Wilmington, DE, USA) equipped with the Agilent HC-C18 column (Santa Clara, USA) with a mobile phase of acetonitrile: water (65:35, v/v).

### Bacterial degradation on fungal HA

For the degradation activity of *B. cereus* No.1 on fungal HA, the standard HA (purity > 98%, Chinese National Compound Library, Shanghai, China) was added to LB medium (50 mL) in flask at 40 μg/mL and bacterial suspension (500 cells/mL) of live No.1 after 24 h-incubation was inoculated into culture medium. Then, this mixture was subsequently incubated at 28℃ in the dark at 150 r/min for 2 days and the culture medium without bacterial addition was used as a control group. The remnant HA and degradation products in the liquid medium were analyzed by UPLC-Q-TOF–MS/MS. The samples were extracted by using equal volume ethyl acetate, subsequently dried and re-dissolved in absolute ethanol and filtered with a 0.45 μm organic phase filter. HPLC was performed on a system equipped with an Agilent HC- C18 column (250 × 4.6 mm, 5 μm) with a mobile phase (acetonitrile: water at 65: 35) at 1 mL/min. High-resolution mass spectral analysis was executed on Agilent 6538A Q-TOF mass spectrometer equipped with an electrospray ion (ESI) source in positive mode. The capillary voltage and fragmentor voltage were maintained at 3.5 kV and 135 V respectively. The flow rate of drying gas was running at 350℃ at 10 L/min. The constituents of samples were detected by using full-scan MS analysis from m/z 50–1700. The collision energy of HA and degradation products was 30 eV.

### Quantitative real-time PCR analysis

RNA samples from the fungal mycelia were obtained using the RNAprep pure Plant Kit (Tiangen, Beijing, China). Specific primers for each gene were designed using Primer Express software (Applied Biosystems, Foster City, CA, USA) and listed in Additional file 1: Table S2. The method of real-time quantitative PCR (RT-qPCR) was based on previous research report [45] and performed using CFX96-C1000 Touch Real-Time PCR Detection System (Bio-Rad, Hercules, CA, USA). The relative transcript levels were determined according to the 2^−ΔΔCt^ method described by Zhang et al. (2016) [46].

## Statistical analysis

Student's *t*-test was used as a significance test to compare the means between two groups. One-way analysis of variance (ANOVA) was performed using statistical analysis system (SAS 9.2, Cary, USA) to assess the significance of differences of the means among multiple groups. All results are expressed as Mean ± Standard Deviation (SD). The level of significance was set at *p* < 0.05.

## Supplementary Information

**Additional file 1: Table S1.** The analysis of VOCs from strain No.1 by headspace solid phase microextraction-gas chromatography-mass spectrometry (HS–SPME–GC–MS). **Table S2.** Primers and relevant information of reference and target genes. F: forward primer, R: reverse primer. **Fig. S1.** (**A**) Macroscopic colony appearance of No.1 strain on LB agar plate for 24 h. (**B**) Phylogenic tree of *Bacillus cereus* No.1 was built by using Clustal W in MEGA software (Molecular Evolutionary Genetics Analysis, version 7.0), with distance options according to the Kimura two-parameter model and clustering with the neighbor-joining (NJ) method. The type strain of the type species, *B. subtilis* ATCC 6051^T^ (CP003329.1), was used as an outgroup. Type strains are indicated by the superscript^T^. Bootstrap values higher than 50% (of 1000 cycles) were indicated. Scale bar indicated substitutions per nucleotide position

## Data Availability

All data generated or analyzed during this study are included in this published article and its Additional files.
