# Supplementary material for: Contrasting regulation of live Bacillus cereus No.1 and its volatiles on Shiraia perylenequinone production
Source: Microb Cell Fact. 2022 Aug 23;21:172. doi: 10.1186/s12934-022-01897-z (PMC9396862; doi:10.1186/s12934-022-01897-z)
Supplement: Supplementary file 1 — Additional file 1: Table S1. The analysis of VOCs from strain No.1 by headspace solid phase microextraction-gas chromatography-mass spectrometry (HS–SPME–GC–MS). Table S2. Primers and relevant information of reference and target genes. F: forward primer, R: reverse primer. Fig. S1. (A) Macroscopic colony appearance of No.1 strain on LB agar plate for 24 h. (B) Phylogenic tree of Bacillus cereus No.1 was built by using Clustal W in MEGA software (Molecular Evolutionary Genetics Analysis, version 7.0), with distance options according to the Kimura two-parameter model and clustering with the neighbor-joining (NJ) method. The type strain of the type species, B. subtilis ATCC 6051T (CP003329.1), was used as an outgroup. Type strains are indicated by the superscriptT. Bootstrap values higher than 50% (of 1000 cycles) were indicated. Scale bar indicated substitutions per nucleotide position [file 12934_2022_1897_MOESM1_ESM.pdf]

Additional file 1

Contrasting regulation of live Bacillus cereus No.1 and its volatiles on *Shiraia* perylenequinone production

Rui Xu, Xin Ping Li, Xiang Zhang, Wen Hao Shen, Chun Yan Min and Jian Wen Wang

✉ Jian Wen Wang

jwwang@suda.edu.cn; bcjwwang@gmail.com

Table S1 The analysis of VOCs from strain No.1 by headspace solid phase microextraction-gas chromatography-mass spectrometry (HS-SPME-GC-MS)

Compound [a]	Identity (%) [b]	Peak area (%) [c]	RT [d]	MW [e]	MF [f]
acetone	5	1.24	1.494	58.08	C_3H_6O
acetic acid	37	0.58	1.812	60.05	$C_2H_4O_2$
2-methyl-1-propanol	38	1.50	2.251	74.12	$C_4H_{10}O$
propane	4	0.73	2.588	44.1	C_3H_8
2-methyl-butanal	64	0.70	2.702	86.13	$C_5H_{10}O$
methyl thiolacetate	9	1.04	3.18	90.14	C_3H_6OS
3-methyl-1-butanol	45	0.57	3.778	88.15	$C_5H_{12}O$
pentanal	33	0.57	3.867	86.13	$C_5H_{10}O$
1,3,5-cycloheptatriene	9	0.53	4.516	92.14	C_7H_8
hexanal	59	1.39	5.247	100.16	$C_6H_{12}O$
2-(methylthio)-ethanol	90	0.93	6.278	92.16	C_3H_8OS
(E)-2-nonen-1-ol	27	1.71	9.37	142.24	$C_9H_{18}O$
S-methyl 3-methylbutanethioate	64	7.51	9.745	132.22	$C_6H_{12}OS$
1,3-trans-5-trans-octatriene	78	0.99	10.089	128.21	$C_8H_{16}O$
3-ethyl-2,5-dimethyl-pyrazine	50	0.58	12.703	108.18	C_8H_{12}
phenylethyl alcohol	74	0.55	14.841	122.16	$C_8H_{10}O$
2,2-dimethyl-pentane	12	0.89	29.804	100.2	C_7H_{16}
2,2-dimethyl-butane	9	0.79	30.021	86.18	C_6H_{14}
2-propenamide	9	0.81	32.502	71.08	C_3H_5NO
2,5,8-triphenyl benzotristriazole	7	0.80	33.806	429.44	$C_{24}H_{15}N_9$
heptadecyl ester heptadecanoic acid	12	0.93	35.524	508.9	$C_{34}H_{68}O_2$
butyl 2-ethylhexyl ester-1,2-benzenedicarboxylic acid	56	1.23	37.687	334.45	$C_{20}H_{30}O_4$

[a] The tested compounds with a relative area of less than 1.0% or less than 70% identity are listed.
[b] Spectra similarity of analyte compounds with those available in the spectral library (NIST).
[c] Relative area of detected compounds as a percentage in reference to the total spectra peaks.
[d] Retention time (RT) of the compounds in the GC-MS analysis.
[e] Molecular weight (MW) of detected compounds.
[f] Molecular formula (MF) of identified compounds.

Table S2 Primers and relevant information of reference and target genes. F: forward primer, R: reverse primer.

Genes symbol	Gene name	Sequence
18S	Reference gene	F: GAAAGTTAGGGGATCGAAGA<br>R: TAGTCGGCATAGTTTACGGT
PKS	Polyketide synthase	F: TGCTGAGGTAGCAGTCAAGC<br>R: TTATGCTACGGTCGTCGCTC
FAD	FAD/FMN-containing dehydrogenase	F: TGTGACCGCCATCACCTTAC<br>R: TTGTCGTATGGGTGGGAAGC
MCO	Multicopper oxidase	F: TATGGCGCTACGAGTGGAC<br>R: ACTCCCTGGCCGATAACGTA
Omef	O-methyltransferase	F: GAACTACCTGAAGGCACGCT<br>R: GCTCGGAAGGATACTCGCTC
ZFTF	Zinc finger transcription factor	F: GAACACCGTCGCAAGATTCG<br>R: TCATTGGCATCGCTTGGAGT
Mono	Salicylate 1-monooxygenase	F: TCTCGGGGAATTATGGCACG<br>R: ACAACCGTTCTCGCATCAGT
ABC	ATP-binding cassette	F: GACTTGAGCCTATCCGCCTC<br>R: AGAGTCGCCTCTGTGATCCT

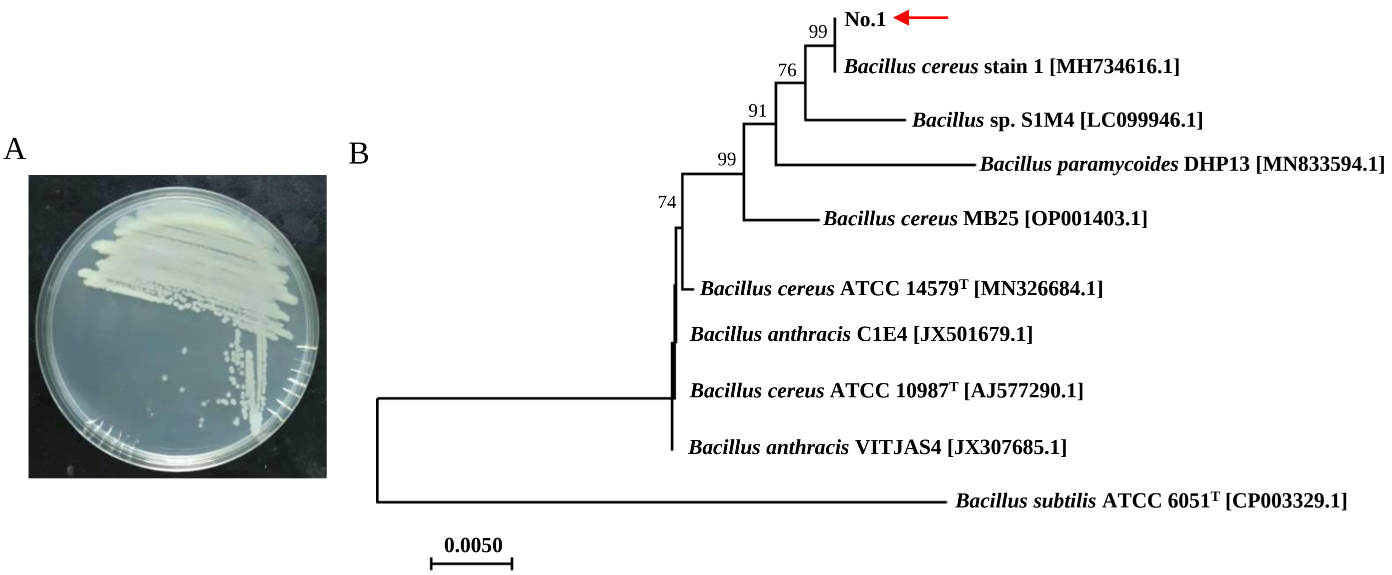

Fig. S1 (A) Macroscopic colony appearance of No.1 strain on LB agar plate for 24 h. (B) Phylogenic tree of *Bacillus cereus* No.1 was built by using Clustal W in MEGA software (Molecular Evolutionary Genetics Analysis, version 7.0), with distance options according to the Kimura two-parameter model and clustering with the neighbor-joining (NJ) method. The type strain of the type species, *B. subtilis* ATCC 6051^T (CP003329.1), was used as an outgroup. Type strains are indicated by the superscript^T. Bootstrap values higher than 50% (of 1000 cycles) were indicated. Scale bar indicated substitutions per nucleotide position.
